# ANCA-Associated Glomerulonephritis: Risk Factors for Renal Relapse

**DOI:** 10.1371/journal.pone.0165402

**Published:** 2016-12-14

**Authors:** Arda Göçeroğlu, Annelies E. Berden, Marta Fiocco, Oliver Floßmann, Kerstin W. Westman, Franco Ferrario, Gill Gaskin, Charles D. Pusey, E. Christiaan Hagen, Laure-Hélène Noël, Niels Rasmussen, Rüdiger Waldherr, Michael Walsh, Jan A. Bruijn, David R. W. Jayne, Ingeborg M. Bajema, on behalf of the European Vasculitis Society (EUVAS)

**Affiliations:** 1 Department of Pathology, Leiden University Medical Center, Leiden, Netherlands; 2 Medical Statistics and Bioinformatics, Leiden University Medical Center, Leiden, Netherlands; 3 Institute of Mathematics, Leiden University, Leiden, Netherlands; 4 Renal Unit, Royal Berkshire Hospital, Reading, United Kingdom; 5 Department of Nephrology, University Hospital Malmö, Malmö, Sweden; 6 Nephropathology Center, San Gerardo Hospital, Monza, Italy; 7 Department of Renal Medicine, Hammersmith Hospital, Imperial College Healthcare NHS Trust, London, United Kingdom; 8 Department of Nephrology, Meander Medical Center, Amersfoort, Netherlands; 9 Department of Pathology, Necker Hospital, René Descartes University, Paris, France; 10 Department of Autoimmune Serology, Statens Seruminstitut, Copenhagen, Denmark; 11 Department of Pathology, University of Heidelberg, Heidelberg, Germany; 12 Department of Medicine (Nephrology), St Joseph’s Hospital, McMaster University, Hamilton, Canada; 13 Department of Clinical Epidemiology & Biostatistics, St Joseph’s Hospital, McMaster University, Hamilton, Canada; 14 Lupus and Vasculitis Clinic, Addenbrooke’s Hospital, Cambridge, United Kingdom; Nippon Medical School, JAPAN

## Abstract

Relapse in ANCA-associated vasculitis (AAV) has been studied previously, but there are few studies on renal relapse in particular. Identifying patients at high risk of renal relapse may aid in optimizing clinical management. We investigated which clinical and histological parameters are risk factors for renal relapse in ANCA-associated glomerulonephritis (AAGN). Patients (n = 174) were newly diagnosed and had mild–moderate or severe renal involvement. Data were derived from two trials of the European Vasculitis Society: MEPEX and CYCAZAREM. The Cox regression model was used to identify parameters increasing the instantaneous risk (= rate) of renal relapse (useful for instant clinical decisions). For identifying predictors of renal relapse during follow-up, we used Fine & Gray’s regression model. Competing events were end-stage renal failure and death. The cumulative incidence of renal relapse at 5 years was 9.5% (95% CI: 4.8–14.3%). In the Cox model, sclerotic class AAGN increased the instantaneous risk of renal relapse. In Fine & Gray’s model, the absence of interstitial infiltrates at diagnosis was predictive for renal relapse. In this study we used two different models to identify possible relationships between clinical and histopathological parameters at time of diagnosis of AAV with the risk of experiencing renal relapse. Sclerotic class AAGN increased the instantaneous risk of renal relapse. This association is most likely due to the high proportion of sclerosed glomeruli reducing the compensatory capacity. The absence of interstitial infiltrates increased the risk of renal relapse which is a warning sign that patients with a relatively benign onset of disease may also be prone to renal relapse. Renal relapses occurring in patients with sclerotic class AAGN and renal relapses occurring in patients without interstitial infiltrates were mutually exclusive, which may indicate that they are essentially different.

## Introduction

Granulomatosis with polyangiitis (GPA) and microscopic polyangiitis (MPA) are the major subtypes of anti-neutrophil cytoplasmic antibody (ANCA)-associated vasculitis (AAV). Approximately 80% of patients with GPA and 90% with MPA develop kidney involvement during the disease course.[1] ANCA-associated glomerulonephritis (AAGN) progresses to end-stage renal failure (ESRF) in approximately 20–40% of patients.[2–5] The gold standard for establishing AAGN is a renal biopsy, which typically shows a pauci-immune necrotizing crescentic glomerulonephritis,[6,7] which can be grouped into four classes.[8] Relapse in ANCA-associated vasculitis has been studied previously, but there are few studies on renal relapse in particular. It is important to find a balance between the risk of relapse and the risk of treatment-related adverse effects.

Identifying patients at high risk of renal relapse may aid in optimizing clinical management. Previous relevant studies mainly focused on relapse in general with clinical data,[2,5,9–19] identifying proteinase 3 (PR3)-ANCA, GPA, lung or cardiovascular involvement, and better renal function at presentation as associated with relapse in general.[2,10–14,16,17,19] Note that different statistical analyses were used in these reports to determine the influence of various parameters on relapse; some published studies employed Fine & Gray’s regression model while others used the standard Cox regression model. Both models are correct but address different research questions.

If more than one endpoint can occur, a competing risk analysis must be performed. In the case of renal relapse, ESRF and death are competing events, because the occurrence of one of them preclude the occurrence of renal relapse. Fine & Gray’s regression model is used to estimate the effect of a risk factor on the cumulative incidence of renal relapse (CIR), which denotes the probability of experiencing renal relapse before time *t*. The classical Cox regression model is used to investigate the effect of risk factors on the rate of renal relapse. The parameters in the Cox regression model are hazard ratios and the interpretation is the traditional one. Note that relationships between risk factors (or explanatory parameters) and cause-specific hazards do not lead to simple relationships between explanatory variables and cumulative incidence. It is important to emphasize that both approaches are valid but that they answer different research questions and may render different results. Thus the effect of a parameter on the CIR might be different from its effect on the rate of renal relapse. Estimation based on Fine & Gray’s model is useful for making predictions from the start of the disease, whereas the rate looks at parameters that increase the instantaneous risk of renal relapse and is useful for instant clinical decisions. In the present study, we apply both methods and discuss implications from their results.

We investigated whether diagnostic clinical and histological parameters are associated with renal relapse in patients with AAV with primary renal involvement. The study aim was to identify diagnostic tools that may be helpful in monitoring and managing patients with AAV, in particular in relation to renal relapse.

## Material and Methods

### Patients

Patients included in this study were newly diagnosed with AAV with either mild to moderate or severe renal involvement (serum creatinine ≤ or > 500 μmol/L (≤ or > 5.8 mg/dl)). Patients were derived from two international multicenter randomized clinical European Vasculitis Society (EUVAS) trials: MEPEX and CYCAZAREM.[11,20] Inclusion criteria for both trials are described elsewhere.[11,20] The diagnosis was based on a clinical presentation compatible with ANCA-associated vasculitis and substantiated by a positive ANCA serology and/or histology.

The MEPEX and CYCAZAREM trial follow-up continued until 12 and 18 months after diagnosis, respectively. During the trials, patients received protocolized treatment regimens.[11,20] After these follow-up periods, patients were treated according to their local physician’s standards. Patients were included and followed-up in the period June 1, 1995, through 30 November, 2006. Patients were included in this study only if histological data obtained from renal biopsy at the time of study entry, clinical data, and long-term follow-up data were available.

Disease definitions were adapted from the Chapel Hill Consensus Conference on the Nomenclature of Systemic Vasculitis and a previous European Union Study.[21,22] Both trials were conducted according to the 1964 Declaration of Helsinki and subsequent amendments. The trials were approved by the local ethics committees of the participating centers throughout Europe. All patients gave written informed consent. The ethics for the use of the data and material for subsequent studies, including this study, was approved by the West Midlands Multi-centre Research Ethics Committee, date 22/09/2004 (reference number: MREC/98/7/37). In addition, this study was performed according to the 'Netherlands Code of Conduct for Scientific Practice', an ethical code for performing observational studies with patient material approved by the Federatie van Medisch Wetenschappelijke Verenigingen (translated: Federation of Medical Scientific Organisations) together with the legal and ethical committee of the Koninklijke Nederlandse Akademie van Wetenschappen (translated: Royal Dutch Academy of Science) and the Nederlandse Organisatie voor Wetenschappelijk Onderzoek (translated: Dutch Organisation for Scientific Research). The data of the patients were analyzed anonymously.

### Clinical and histological parameters

Candidate parameters for clinical predictors of renal relapse in this study were serum creatinine levels, age, sex, diagnosis (GPA or MPA), ANCA-antigen specificity (PR3-ANCA or myeloperoxidase (MPO)-ANCA), and receiving plasma exchange during induction therapy. Patients were subdivided into two groups of GPA and MPA based on the clinical criteria. Renal-limited vasculitis was regarded as a form of MPA.

Candidate parameters for histological predictors were determined from paraffin sections of renal biopsies. Stains used for evaluation were silver, periodic acid–Schiff, hematoxylin and eosin, and trichrome. Sections were reviewed by two of a panel of five participating pathologists (IMB, FF, LHN, RW, and/or JAB). Both pathologists, blinded to patient data and the other observer’s results, scored the biopsies separately and according to a previously standardized protocol, which was proven to be comprehensive and reproducible when used for histologic analysis.[23] One of the histological parameters included in this previously standardized protocol is interstitial infiltrates. Interstitial infiltrates were scored according to the following categories:

- None: <10% of the unscarred parenchyma infiltrated.
- Mild: 10 to 25% of the unscarred parenchyma infiltrated.
- Quite dense: 26 to 50% of the unscarred parenchyma infiltrated.
- Very dense: >50% of the unscarred parenchyma infiltrated.

In this study, only biopsies with a minimum of seven whole glomeruli were analyzed for glomerular lesions and the histopathological classification system of AAGN.[8] During plenary meetings, the panel of five pathologists decided upon the final scores to achieve consensus for each biopsy.

### Clinical outcomes

The clinical outcome parameter was first renal relapse. A renal relapse was defined as a rise in serum creatinine of >30% or a fall in estimated glomerular filtration rate >25% and/or new hematuria or proteinuria (all attributable to active vasculitis), as indicated by the Birmingham Vasculitis Activity Score.[24–26] Patients were followed up until the last visit or death.

### Statistical analyses

In this study, more than one endpoint could occur, namely renal relapse, ESRF, or death. The event of interest was renal relapse, while ESRF and death were competing events. Two regression models used in the competing risks framework were estimated here: Fine & Gray’s model and Cox model. To study the effect of risk factors on the CIR the former model is employed while the latter is used to study the effects of risk factors on the rate of renal relapse, i.e. the cause-specific hazard. For more details concerning the difference between the two models, see Andersen *et al*. and Koller *et al*.[27,28] The technical aspects of competing risks were described previously by Putter *et al*. [29]

Univariate analyses with both methods were performed on every clinical and histological parameter. These analyses were performed on all patients without ESRF at baseline (n = 149), except for glomerular lesions and the histopathological class of AAGN. In addition, we performed a χ2-test to see whether the percent of renal relapse differed significantly between the histopathological classes. Also a Pearson correlation test was performed to investigate whether the presence of interstitial infiltrate was correlated with interstitial fibrosis. All baseline parameters were included as fixed covariates.

Because of the number of parameters (13 in this study) and the relatively low number of events, inclusion of too many parameters carries the risk of “overfitting.”[30] Therefore, predefined smaller sets of entry parameters were included in the multivariate analyses, as follows: based on the original publication of the histopathological classification system of AAGN[8]; based on parameters described previously more than once as being associated with relapse; and based on only histological parameters.

We denoted hazard ratios estimated by employing Cox regression model as cause-specific hazard ratio (csHR) and the hazard ratios estimated by using Fine & Gray’s regression model as Fine & Gray’s HR (F&G HR). All hazard ratios are provided with 95% confidence intervals (CI). A *P* value of less than 0.05 was considered significant. Statistical analyses were performed in SPSS (version 20.0; SPSS Inc, Chicago, IL) and R 2–18 (http://cran.r-project.org). All analyses concerning competing risks were performed with the mstate library.[31,32]

## Results

### Patients

A total of 174 patients with newly diagnosed AAV and a renal biopsy at diagnosis were included in this study. Table 1 shows the baseline patient characteristics. The median follow-up time was 102 months (range: 38–136 months).

**Table 1:** Baseline characteristics of all patients.

Characteristic		Value
Number of patients		174
Age (years)		60.3 ± 13.1^a^
Male		94 (54)
Diagnosis		
	GPA	74 (43)
	MPA	100 (57)
ANCA antigen		
	PR3	81 (47)
	MPO	80 (46)
	Negative	7 (4)
	Double positive	3 (2)
	NR	3 (2)
Serum creatinine		
	≤ 100 μmol/L	23 (13)
	101–200 μmol/L	23 (13)
	>201 μmol/L	128 (74)
ESRF at baseline		25 (14)
PLEX therapy		
	Yes	46 (26)
	No	128 (74)
Histopathological class^b^		
	Focal	23 (20)
	Crescentic	58 (51)
	Mixed	18 (16)
	Sclerotic	14 (12)

Data are presented as n (%) unless otherwise noted.

Abbreviations: ANCA, anti-neutrophil cytoplasmic antibody; ESRF, end-stage renal failure; GPA, granulomatosis with polyangiitis; MPA, microscopic polyangiitis; MPO, myeloperoxidase; NR, not reported/not performed; PLEX, plasma exchange therapy; PR3, proteinase 3.

^a^Mean (SD).

^b^Only patients with at least 7 whole glomeruli in their renal biopsy and no ESRF at baseline.

### Renal relapse

**Fig 1:**
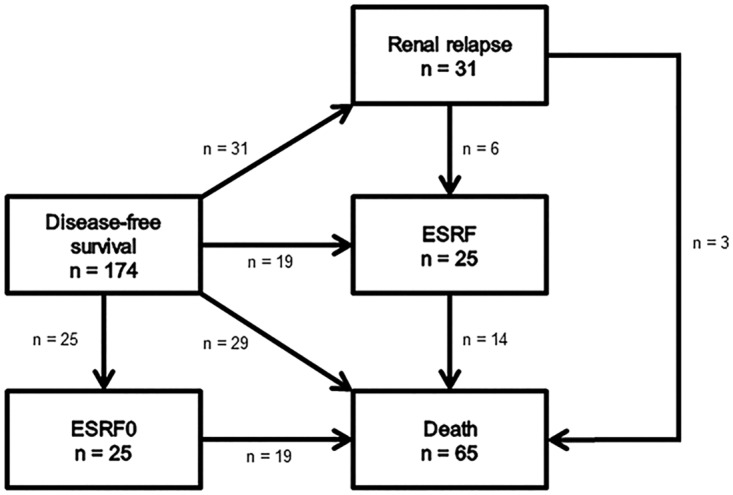
Events. Overview of different events experienced by 174 patients during follow-up. Twenty-five patients presented with ESRF at baseline. Nineteen of them died during follow-up. Thirty-one patients experienced renal relapse during follow-up; six of them developed ESRF, of whom four died, and three died without ESRF during follow-up. Nineteen patients developed ESRF without renal relapse (competing event 1), of whom 10 died at a later timepoint. Twenty-nine patients died without experiencing renal relapse or ESRF (competing event 2). Seventy patients experienced no event during follow-up. Abbreviations: DSF, disease-free survival; ESRF, end-stage renal failure; ESRF0, end-stage renal failure at baseline.

**Fig 2:**
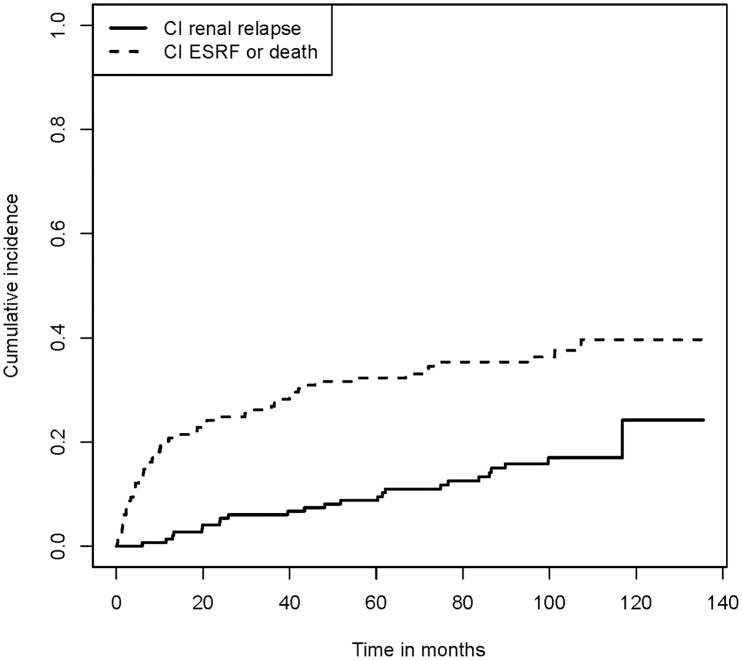
Cumulative incidence of renal relapse, end-stage renal failure or death. Cumulative incidence of patients who experienced renal relapse (event of interest) and patients who developed ESRF or died (competing events). This figure illustrates the probability of experiencing a renal relapse and the probability of developing ESRF or dying without experiencing a renal relapse. Abbreviations: CI, cumulative incidence; ESRF, end-stage renal failure.

Of the 174 patients, 25 could not experience a renal relapse because they had ESRF at baseline. Of the remaining 149 patients, 22 were chronic kidney disease (CKD) stage 1–2 and 127 were CKD stage 3–5. Of these 22 CKD stage 1–2 patients, 5 (22.7%) developed CKD stage 3–5 within 5 years of follow-up. None of these 5 had experienced a renal relapse. In total, 31 patients experienced a renal relapse during follow-up. The CIR at 5 years was 9.5% (95% CI: 4.8–14.3%). A total of 19 patients developed ESRF during follow-up without experiencing renal relapse, and 29 died without experiencing renal relapse and without developing ESRF during follow-up. All patients who died during the follow-up period had CKD stage ≥ 3 at baseline. Seventy patients had none of these events during follow-up (Figs 1 and 2).

**Fig 3:**
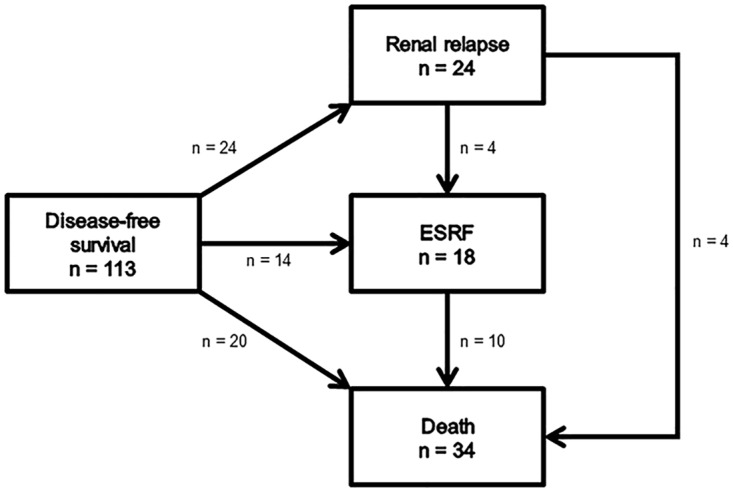
Events of the 113 patients with ≥7 glomeruli in their renal biopsy without end-stage renal failure at baseline. Twenty-four patients experienced a renal relapse during follow-up. Of these 24 patients, four developed ESRF, of which three died, and four died without ESRF during follow-up. Fourteen patients developed ESRF without renal relapse (competing event 1), of whom 7 died at a later timepoint. Twenty patients died without renal relapse and without ESRF (competing event 2). Fifty-five patients experienced no event during follow-up. Abbreviations: DSF, disease-free survival; ESRF, end-stage renal failure.

Of the 149 patients, 113 had adequate renal tissue samples (at least seven whole glomeruli in the renal biopsy) for classification purposes. Their diagnostic renal biopsies were classified as follows: 23 focal class (20.4%), 58 crescentic class (51.3%), 18 mixed class (15.9%), and 14 sclerotic class (12.4%) (Table 2). Of these 113 patients, 24 experienced a renal relapse during follow-up. The numbers of patients having a renal relapse per class were 5/23 (21.7%) focal class, 9/58 (15.5%) crescentic class, 4/18 (22.2%) mixed class, and 6/14 (42.9%) sclerotic class (χ2-test: P = 0.167). The distribution of patient ages did not differ across classes. In particular, patients from the sclerotic class were not older than those in other classes. During the trials, therapies given to the patients did not differ among the four classes. Fourteen patients developed ESRF during follow-up without experiencing renal relapse (competing event 1). Twenty patients died without experiencing renal relapse and without developing ESRF during follow-up (competing event 2) (Fig 3).

**Table 2:** Baseline characteristics of patients per histopathological class.

Characteristic		Value per class			
		Focal class	Crescentic class	Mixed class	Sclerotic class
Number of patients		23	58	18	14
Age (years)		55.4 ± 13.8^a^	60.4 ± 13.6^a^	59.8 ± 9.2^a^	63.8 ± 12.3^a^
Male		14 (61)	28 (48)	11 (61)	6 (43)
Diagnosis					
	GPA	16 (70)	25 (43)	6 (33)	3 (21)
	MPA	7 (30)	33 (57)	12 (67)	11 (79)
ANCA antigen					
	PR3	17 (74)	28 (48)	8 (44)	3 (21)
	MPO	5 (22)	25 (43)	9 (50)	11 (79)
	Negative	0 (0)	2 (4)	1 (6)	0 (0)
	Double positive	1 (4)	2 (4)	0 (0)	0 (0)
	NR	0 (0)	1 (2)	0 (0)	0 (0)
Serum creatinine					
	≤ 100 μmol/L	14 (61)	1 (2)	1 (6)	0 (0)
	101–200 μmol/L	3 (13)	7 (12)	3 (17)	3 (21)
	>201 μmol/L	6 (26)	50 (86)	14 (78)	11 (79)
PLEX therapy					
	Yes	1 (4)	18 (69)	5 (28)	4 (29)
	No	22 (96)	40 (31)	13 (72)	10 (71)

Data are presented as n (%) unless otherwise noted.

Sample size: 113 patients (patients with at least 7 whole glomeruli in their renal biopsy and no end-stage renal failure at baseline).

Abbreviations: ANCA, anti-neutrophil cytoplasmic antibody; GPA, granulomatosis with polyangiitis; MPA, microscopic polyangiitis; MPO, myeloperoxidase; NR, not reported/not performed; PLEX, plasma exchange therapy; PR3, proteinase 3.

^a^Mean (SD).

### Competing risks analyses

To investigate which parameters are associated with the CIR, Fine & Gray’s regression model was used. The traditional Cox regression model was used to estimate the effect of the parameters on the rate of renal relapse.[27]

### Fine & Gray’s regression model

The univariate analyses showed that age, interstitial infiltrates, and intra-epithelial infiltrates were associated with CIR (Table A in S1 File). Older age was associated with a lower risk for experiencing a renal relapse. Higher scores of interstitial infiltrates and intra-epithelial infiltrates, which are both signs of acute disease activity, were associated with a lower risk for renal relapse. The histopathological class and CKD stage were not significantly associated with the risk of renal relapse.

Among all histological parameters, only interstitial infiltrates had a significant association on the risk of renal relapse (the CIR) in the multivariate analysis (Table 3). Although there was a correlation between interstitial infiltrates and interstitial fibrosis, this correlation was relatively weak (r = 0.236, P = 0.004). Therefore, interstitial fibrosis was not predictive for renal relapse. Patients with mild infiltrates had a four times lower risk for renal relapse than patients without interstitial infiltrates (F&G HR: 0.09; 95% CI: 0.02–0.39; *P* = 0.001). This association persisted when correcting for the patient cohort indicator.

**Table 3:** Multivariate analysis with Fine & Gray’s model based on histological parameters.

Parameter		Renal relapse	
		P Value	F&G HR (95% CI)
Interstitial infiltrates			
	None	-	1
	Mild	0.001	0.09 (0.02–0.39)
	Quite dense	0.1	0.16 (0.02–1.56)
	Very dense	0.5	0.29 (0.01–7.83)
Interstitial fibrosis			
	None	-	1
	Focal	0.9	1.06 (0.24–4.67)
	Diffuse	0.7	1.59 (0.19–13.44)
Tubular atrophy			
	None	-	1
	Small foci	0.7	1.44 (0.23–8.85)
	Extensive	0.9	0.87 (0.05–14.34)
Intra-epithelial infiltrates^a^		0.2	0.34 (0.05–2.08)
Histopathological class			
	Focal	0.2	0.21 (0.02–2.11)
	Crescentic	0.3	0.40 (0.08–2.00)
	Mixed	0.9	1.06 (0.18–6.11)
	Sclerotic	-	1

Sample size: 112 patients

Abbreviations: 95% CI, 95% confidence interval; NS, not significant; F&G HR, Fine and Gray’s hazard ratio.

^a^Reference group: No intra-epithelial infiltrates.

Among all clinical parameters, only age, corrected for other parameters, was associated with patients experiencing a renal relapse (Table B in S1 File).

### Cox regression model for renal relapse

Among all baseline parameters, only the histopathological class was a significant risk factor for renal relapse in the univariate analyses (Table C in S1 File). CKD stage was not associated with renal relapse. After correction for age, baseline serum creatinine, and plasma exchange therapy, the histopathological class remained the only statistical significant risk factor for experiencing a renal relapse (Table 4). Focal and crescentic class biopsies were associated with a lower cause-specific hazard ratio compared to sclerotic class biopsies. Focal class had a 10.1 times lower rate than the sclerotic class (csHR: 0.10; 95% CI: 0.02–0.60; *P* = 0.01), and crescentic class had a 4.7 times lower rate than the sclerotic class (csHR: 0.21; 95% CI: 0.07–0.62; *P* = 0.004). Patient cohort (MEPEX or CYCAZAREM) did not affect these associations.

**Table 4:** Multivariate analyses with both models based on the original publication of the histopathological classification system of ANCA-associated glomerulonephritis (Berden et al, 2010 [8]).

Parameter		Renal relapse			
		Cox regression model		Fine & Gray’s model	
		P Value	csHR (95% CI)	P Value	F&G HR (95% CI)
Serum creatinine					
	≤ 100 μmol/L	-	1	-	1
	101–200 μmol/L	0.6	0.65 (0.11–3.70)	0.6	0.58 (0.07–4.66)
	>201 μmol/L	0.4	0.45 (0.08–2.43)	0.4	0.42 (0.05–3.80)
Age		0.3	0.98 (0.96–1.01)	0.02	0.97 (0.94–0.996)
Plasma exchange therapy^a^		0.9	1.10 (0.33–3.69)	0.8	0.85 (0.23–3.14)
Histopathological class					
	Focal	0.01	0.10 (0.02–0.60)	0.2	0.24 (0.03–2.19)
	Crescentic	0.004	0.21 (0.07–0.62)	0.07	0.34 (0.10–1.09)
	Mixed	0.08	0.31 (0.08–1.15)	0.6	0.68 (0.18–2.58)
	Sclerotic	-	1	-	1

Sample size: 113 patients

Abbreviations: 95% CI, 95% confidence interval; csHR, cause-specific hazard ratio; NS, not significant; F&G HR, Fine and Gray’s hazard ratio.

^a^Reference group: No plasma exchange therapy received.

With the inclusion of histopathological class, baseline serum creatinine, ANCA type, and diagnosis, only the histopathological class was a significant risk factor for renal relapse (Table 5). Again, focal and crescentic classes were associated with a lower rate compared to sclerotic class. In this model, focal class had a 10.8 times lower rate than the sclerotic class (csHR: 0.09; 95% CI: 0.02–0.55; *P* = 0.009), and crescentic class had a 4.8 times lower hazard rate than the sclerotic class (csHR: 0.21; 95% CI: 0.07–0.64; *P* = 0.006). There was no effect of patient cohort on these associations.

**Table 5:** Multivariate analyses with both models based on previously described parameters associated with relapse.

Parameter		Renal relapse			
		Cox regression model		Fine & Gray’s model	
		P Value	csHR (95% CI)	P Value	F&G HR (95% CI)
Serum creatinine					
	≤ 100 μmol/L	-	1	-	1
	101–200 μmol/L	0.6	0.62 (0.12–3.33)	0.5	0.44 (0.05–3.62)
	>201 μmol/L	0.4	0.46 (0.08–2.65)	0.2	0.23 (0.02–2.61)
Diagnosis^a^		0.2	0.55 (0.20–1.51)	0.9	0.96 (0.24–3.87)
PR3-ANCA^b^		0.6	0.61 (0.09–4.22)	0.3	0.45 (0.10–2.03)
MPO-ANCA^b^		0.9	0.84 (0.14–5.07)	0.4	0.62 (0.21–1.79)
Histopathological class					
	Focal	0.009	0.09 (0.02–0.55)	0.3	0.32 (0.04–2.28)
	Crescentic	0.006	0.21 (0.07–0.64)	0.2	0.46 (0.13–1.63)
	Mixed	0.06	0.26 (0.07–1.07)	0.7	0.73 (0.18–2.98)
	Sclerotic	-	1	-	1

Sample size: 112 patients

Abbreviations: 95% CI, 95% confidence interval; ANCA, anti-neutrophil cytoplasmic antibody; csHR, cause-specific hazard ratio; MPO, myeloperoxidase; NS, not significant; PR3, proteinase 3; F&G HR, Fine and Gray’s hazard ratio.

^a^Reference group: Granulomatosis with polyangiitis.

^b^Reference group: Negative.

## Discussion

This study shows that the histopathological class of AAGN in the renal biopsy at diagnosis is a risk factor for renal relapse. More specifically, sclerotic class was associated with a higher rate of renal relapse during long-term follow-up. It is important to emphasize that the effect of sclerotic class on the risk of renal relapse, i.e. the cumulative incidence, estimated by the Fine & Gray’s model is different from its effects on the rate, i.e. the cause-specific hazard, estimated by the Cox regression proportional hazard model. This is because the way in which risk factors (or explanatory variables) are associated with the cause-specific hazards may not coincide with the way these covariates are associated with the cumulative incidence. The sclerotic class in AAGN is defined by ≥50% globally sclerosed glomeruli, meaning that the majority of glomeruli are non-functioning and that the compensatory ability of the kidneys is relied on heavily. Therefore, in these patients, a renal relapse may become more readily apparent because the compensatory capacity of a sclerotic class kidney is reduced. Moreover, with fewer functioning glomeruli, these glomeruli may become more vulnerable to a second hit, i.e., a relapse. In patients with AAGN that is not in the sclerotic class, minor relapses may remain subclinical because of the relatively higher number of preserved glomeruli and their compensatory ability. Patients’ treatments were not based on the histopathological classification. Therefore, the sclerotic class may provide a setting in which renal relapse may be more likely to be detected than in the setting of another histopathological class.

To investigate the effect of risk factors on the risk of renal relapse, i.e. the cumulative incidence, we applied Fine & Gray’s regression model. Results show that absence of interstitial inflammatory infiltrates is associated with the risk of renal relapse. Patients with these infiltrates had a lower risk for future renal relapse than patients without these inflammatory infiltrates. The association of interstitial infiltrates with renal relapse persisted when corrected for other histological parameters. Previous EUVAS studies focused on predictive clinical and serological parameters for relapse in general. Walsh *et al*. investigated clinical and serological parameters predictive for relapse in general in a European cohort consisting of 535 patients. In that study, PR3-ANCA, lower serum creatinine levels at presentation, cardiovascular involvement, and GPA were independently associated with an increased risk for relapse, whether in the kidney or any other organ.[19] Our study is based on the histopathological data of those patients from the previous study by Walsh *et al*. who had a renal biopsy with sufficient tissue for proper evaluation; thus, we could investigate which histological parameters are predictive for renal relapse. Our finding that the absence of interstitial infiltrates is predictive for renal relapse is in line with the finding by Walsh *et al*. that better renal function increases the risk for a relapse in general because absence of interstitial infiltrates also correlates with better renal function at the time of biopsy.[33,34] Experiencing a renal relapse has a negative influence on renal outcome.[35] Therefore, clinicians should realize that renal relapses must be identified and treated and keep in mind that those patients with a relatively benign clinical course at onset in particular will be prone to developing a renal relapse.

This study shows a higher cumulative risk for ESRF or death compared to renal relapse as shown in Fig 2. These results may have been influenced by the inclusion of patient from the MEPEX trial which included patients with serum creatinine >500 μmol/L or immediate dialysis dependency. Nineteen patients (11%) had ESRF during follow-up without experiencing a (clinical) renal relapse. It is possible that these patients had subclinical renal relapses, but they were not detected clinically. Based on this knowledge, we emphasize the need for chronic kidney disease management and renal protective strategies.

Our study has a number of limitations. Because of the sample size and relatively low number of events, we were limited in the size of the predefined multivariate analyses. To avoid bias, all multivariate analyses were predefined before the start of this study. To use the best possible predefined analyses, we constructed them based on the literature regarding the histopathological classification system of AAGN. Unfortunately, repeat biopsies during the time of renal relapse were not performed because it is generally considered that the risk of taking a biopsy would not weight against the benefit of a histologically proven renal relapse, keeping in mind that these can be diagnosed with a high level of certainty on the basis of the clinical findings.

In conclusion, we used two regression models to identify possible relationships between clinical and histopathological parameters at time of diagnosis of AAV with the risk of renal relapse, i.e. the cumulative incidence, and the effect on the rate of renal relapse, i.e. the cause-specific hazard. The effect of sclerotic class on the risk of renal relapse estimated by the Fine & Gray’s regression model was different from its effects on the rate estimated by the Cox regression model. Most likely, the lack of compensatory function in the largely sclerosed kidneys gives rise to the identification of a relatively high number of renal relapses. A strong predictive parameter for renal relapse was the absence of interstitial infiltrates as determined with the Fine & Gray model. Combining these results with those of previous studies, it seems that the patient with AAV characterized by a relatively benign clinical setting at onset is prone to a renal relapse. In this study, renal relapses occurring in patients with sclerotic class AAGN and renal relapses occurring in patients without interstitial infiltrates were mutually exclusive, which may indicate that they are essentially other kinds of relapses. Further studies are called to look further into the characteristics of renal relapses in AAV, in particular to find out whether the histopathological data at disease onset could serve as a guideline for the management of renal relapses in AAV.

## Supporting Information

S1 File **Table A. Univariate analyses with Fine & Gray’s model**.
Abbreviations: 95% CI, 95% confidence interval; ANCA, anti-neutrophil cytoplasmic antibody; CKD, chronic kidney disease; GPA, granulomatosis with polyangiitis; MPA, microscopic polyangiitis; MPO, myeloperoxidase; NS, not significant PR3, proteinase 3; F&G dHR, Fine and Gray’s hazard ratio.
^a^Reference group: Female.
^b^Reference group: Granulomatosis with polyangiitis.
^c^Reference group: Negative.
^d^Reference group: No plasma exchange therapy received.
^e^Reference group: No intra-epithelial infiltrates.
**Table B. Multivariate analysis with Fine & Gray’s model based on clinical parameters and histopathological class**.
Sample size: 112 patients
Abbreviations: 95% CI, 95% confidence interval; ANCA, anti-neutrophil cytoplasmic antibody; GPA, granulomatosis with polyangiitis; MPA, microscopic polyangiitis; MPO, myeloperoxidase; NS, not significant; PR3, proteinase 3; F&G HR, Fine and Gray’s hazard ratio.
^a^Reference group: Female.
^b^Reference group: Granulomatosis with polyangiitis.
^c^Reference group: Negative.
^d^Reference group: No plasma exchange therapy received.
**Table C. Univariate analyses with Cox regression model**.
Abbreviations: 95% CI, 95% confidence interval; ANCA, anti-neutrophil cytoplasmic antibody; CKD, chronic kidney disease; csHR, cause-specific hazard ratio; GPA, granulomatosis with polyangiitis; MPA, microscopic polyangiitis; MPO, myeloperoxidase; PR3, proteinase 3.
^a^Reference group: Female.
^b^Reference group: Granulomatosis with polyangiitis.
^c^Reference group: Negative.
^d^Reference group: No plasma exchange therapy received.
^e^Reference group: No intra-epithelial infiltrates.
(DOC)
