# Supplementary material for: ANCA-Associated Glomerulonephritis: Risk Factors for Renal Relapse
Source: PLoS One. 2016 Dec 14;11(12):e0165402. doi: 10.1371/journal.pone.0165402 (PMC5156375; doi:10.1371/journal.pone.0165402)
Supplement: S1 File — Table A. Univariate analyses with Fine & Gray’s model. Abbreviations: 95% CI, 95% confidence interval; ANCA, anti-neutrophil cytoplasmic antibody; CKD, chronic kidney disease; GPA, granulomatosis with polyangiitis; MPA, microscopic polyangiitis; MPO, myeloperoxidase; NS, not significant PR3, proteinase 3; F&G dHR, Fine and Gray’s hazard ratio. aReference group: Female. bReference group: Granulomatosis with polyangiitis. cReference group: Negative. dReference group: No plasma exchange therapy received. eReference group: No intra-epithelial infiltrates. Table B. Multivariate analysis with Fine & Gray’s model based on clinical parameters and histopathological class. Sample size: 112 patients Abbreviations: 95% CI, 95% confidence interval; ANCA, anti-neutrophil cytoplasmic antibody; GPA, granulomatosis with polyangiitis; MPA, microscopic polyangiitis; MPO, myeloperoxidase; NS, not significant; PR3, proteinase 3; F&G HR, Fine and Gray’s hazard ratio. aReference group: Female. bReference group: Granulomatosis with polyangiitis. cReference group: Negative. dReference group: No plasma exchange therapy received. Table C. Univariate analyses with Cox regression model. Abbreviations: 95% CI, 95% confidence interval; ANCA, anti-neutrophil cytoplasmic antibody; CKD, chronic kidney disease; csHR, cause-specific hazard ratio; GPA, granulomatosis with polyangiitis; MPA, microscopic polyangiitis; MPO, myeloperoxidase; PR3, proteinase 3. aReference group: Female. bReference group: Granulomatosis with polyangiitis. cReference group: Negative. dReference group: No plasma exchange therapy received. eReference group: No intra-epithelial infiltrates. (DOC) [file pone.0165402.s001.doc]

# ANCA-Associated Glomerulonephritis: Risk Factors for Renal Relapse

**Supplemental information**

# Arda Göçeroğlu^1*^, Annelies E. Berden^1^, Marta Fiocco^2,3^, Oliver Floßmann^4^, Kerstin W. Westman^5^, Franco Ferrario^6^, Gill Gaskin^7^, Charles D. Pusey^7^, E. Christiaan Hagen^8^, Laure-Hélène Noël^9^, Niels Rasmussen^10^, Rüdiger Waldherr^11^, Michael Walsh^12,13^, Jan A. Bruijn^1^, David R.W. Jayne^14^, Ingeborg M. Bajema^1^, on behalf of the European Vasculitis Society (EUVAS)^

^1^ Department of Pathology, Leiden University Medical Center, Leiden, Netherlands

^2^ Medical Statistics and Bioinformatics, Leiden University Medical Center, Leiden, Netherlands

^3^ Institute of Mathematics, Leiden University, Leiden, Netherlands

^4^ Renal Unit, Royal Berkshire Hospital, Reading, United Kingdom

^5^ Department of Nephrology, University Hospital Malmö, Malmö, Sweden

^6^ Nephropathology Center, San Gerardo Hospital, Monza, Italy

^7^ Department of Renal Medicine, Hammersmith Hospital, Imperial College Healthcare NHS Trust, London, United Kingdom

^8^ Department of Nephrology, Meander Medical Center, Amersfoort, Netherlands

^9^ Department of Pathology, Necker Hospital, René Descartes University, Paris, France

^10^ Department of Autoimmune Serology, Statens Seruminstitut, Copenhagen, Denmark

^11^ Department of Pathology, University of Heidelberg, Heidelberg, Germany

^12^ Department of Medicine (Nephrology), St Joseph’s Hospital, McMaster University, Hamilton, Canada

^13^ Department of Clinical Epidemiology & Biostatistics, St Joseph’s Hospital, McMaster University, Hamilton, Canada

^14^ Lupus and Vasculitis Clinic, Addenbrooke’s Hospital, Cambridge, United Kingdom

* Corresponding Author

E-mail: [A.Goeceroglu@lumc.nl](mailto:A.Goeceroglu@lumc.nl) (AG)

**Content:** 3 tables

**Tables**

**Table A. Univariate analyses with Fine & Gray’s model**

| **Parameter** | **Renal relapse** | |
| --- | --- | --- |
|  | **P Value** | **F&G HR (95% CI)** |
| Clinical parameters | | |
| Gender^a^ | 0.9 | 1.04 (0.47-2.30) |
| Serum creatinine  ≤ 100 µmol/L  101-200 µmol/L  >201 µmol/L | -  0.6  0.2 | 1  0.74 (0.24-2.31)  0.51 (0.20-1.32) |
| Age | 0.003 | 0.96 (0.94-0.99) |
| Diagnosis (GPA, MPA)^b^ | 0.4 | 0.73 (0.33-1.62) |
| PR3-ANCA^c^ | 0.4 | 1.43 (0.63-3.24) |
| MPO-ANCA^c^ | 0.4 | 0.73 (0.32-1.64) |
| Plasma exchange therapy^d^ | 0.5 | 0.70 (0.24-2.02) |
| CKD stage | 0.2 | 0.59 (0.26-1.34) |
| Interstitial lesions | | |
| Interstitial infiltrates  None  Mild infiltrate  Quite dense infiltrate  Very dense infiltrate | -  0.006  0.01  0.5 | 1  0.25 (0.09-0.67)  0.26 (0.09-0.72)  0.45 (0.05-3.79) |
| Interstitial fibrosis  None  Focal  Diffuse | -  0.5  0.7 | 1  0.70 (0.27-1.80)  0.81 (0.25-2.64) |
| Tubular lesions | | |
| Tubular atrophy  None  Small foci  Extensive | -  0.1  0.5 | 1  0.47 (0.19-1.16)  0.60 (0.20-2.15) |
| Intra-epithelial infiltrates^e^ | 0.04 | 0.43 (0.20-0.94) |
| Glomerular lesions | | |
| Histopathological class  Focal  Crescentic  Mixed  Sclerotic | 0.7  0.8  0.3  - | 0.99 (0.24-2.48)  (0.29-5.07)  (0.51-6.93)  1 |
| Fibrinoid necrosis | 0.5 | 0.99 (0.97-1.01) |

Abbreviations: 95% CI, 95% confidence interval; ANCA, anti-neutrophil cytoplasmic antibody; CKD, chronic kidney disease; GPA, granulomatosis with polyangiitis; MPA, microscopic polyangiitis; MPO, myeloperoxidase; NS, not significant PR3, proteinase 3; F&G dHR, Fine and Gray’s hazard ratio.

^a^Reference group: Female.

^b^Reference group: Granulomatosis with polyangiitis.

^c^Reference group: Negative.

^d^Reference group: No plasma exchange therapy received.

^e^Reference group: No intra-epithelial infiltrates.

**Table B. Multivariate analysis with Fine & Gray’s model based on clinical parameters and histopathological class**

| **Parameter** | **Renal relapse** | |
| --- | --- | --- |
|  | **P Value** | **F&G HR (95% CI)** |
| Gender^a^ | 0.9 | 1.04 (0.40-2.68) |
| Serum creatinine  ≤ 100 µmol/L  101-200 µmol/L  >201 µmol/L | -  0.6  0.4 | 1  0.55 (0.06-4.65)  0.35 (0.03-4.73) |
| Age | 0.02 | 0.97 (0.94-0.99) |
| Diagnosis (GPA, MPA)^b^ | 0.8 | 0.88 (0.24-3.16) |
| PR3-ANCA^c^ | 0.2 | 0.38 (0.08-1.82) |
| MPO-ANCA^c^ | 0.3 | 0.54 (0.18-1.61) |
| Plasma exchange therapy^d^ | 0.7 | 0.80 (0.21-3.01) |
| Histopathological class  Focal  Crescentic  Mixed  Sclerotic | 0.2  0.1  0.6  - | 0.26 (0.03-2.19)  0.38 (0.11-1.31)  0.72 (0.20-2.68)  1 |

Sample size: 112 patients

Abbreviations: 95% CI, 95% confidence interval; ANCA, anti-neutrophil cytoplasmic antibody; GPA, granulomatosis with polyangiitis; MPA, microscopic polyangiitis; MPO, myeloperoxidase; NS, not significant; PR3, proteinase 3; F&G HR, Fine and Gray’s hazard ratio.

^a^Reference group: Female.

^b^Reference group: Granulomatosis with polyangiitis.

^c^Reference group: Negative.

^d^Reference group: No plasma exchange therapy received.

**Table C. Univariate analyses with Cox regression model**

| **Parameter** | **Renal relapse** | |
| --- | --- | --- |
|  | **P Value** | **csHR (95% CI)** |
| Clinical parameters | | |
| Gender^a^ | 0.3 | 1.53(0.75-3.13) |
| Serum creatinine  ≤ 100 µmol/L  101-200 µmol/L  >201 µmol/L | -  0.9  0.6 | 1  1.00 (0.32-3.12)  1.30 (0.52-3.28) |
| Age | 0.4 | 0.99 (0.97-1.02) |
| Diagnosis (GPA, MPA)^b^ | 0.3 | 0.68 (0.33-1.40) |
| PR3-ANCA^c^ | 0.6 | 1.26 (0.60-2.64) |
| MPO-ANCA^c^ | 0.8 | 0.90 (0.43-1.89) |
| Plasma exchange therapy^d^ | 0.6 | 1.28 (0.52-3.16) |
| CKD stage | 0.5 | 0.77 (0.33-1.78) |
| Interstitial lesions | | |
| Interstitial infiltrates  None  Mild infiltrate  Quite dense infiltrate  Very dense infiltrate | -  0.08  0.2  0.5 | 1  0.43 (0.16-1.11)  0.50 (0.18-1.36)  1.96 (0.23-16.83) |
| Interstitial fibrosis  None  Focal  Diffuse | -  0.7  0.5 | 1  1.21 (0.48-3.06)  1.46 (0.49-4.35) |
| Tubular lesions | | |
| Tubular atrophy  None  Small foci  Extensive | -  0.5  0.9 | 1  0.75 (0.33-1.72)  1.04 (0.34-3.19) |
| Intra-epithelial infiltrates^e^ | 0.1 | 0.58 (0.29-1.19) |
| Glomerular lesions | | |
| Histopathological class  Focal  Crescentic  Mixed  Sclerotic | 0.008  0.006  0.1  - | 0.20 (0.06-0.66)  0.24 (0.08-0.66)  0.36 (0.10-1.28)  1 |
| Fibrinoid necrosis | 0.3 | 0.99 (0.97-1.01) |

Abbreviations: 95% CI, 95% confidence interval; ANCA, anti-neutrophil cytoplasmic antibody; CKD, chronic kidney disease; csHR, cause-specific hazard ratio; GPA, granulomatosis with polyangiitis; MPA, microscopic polyangiitis; MPO, myeloperoxidase; PR3, proteinase 3.

^a^Reference group: Female.

^b^Reference group: Granulomatosis with polyangiitis.

^c^Reference group: Negative.

^d^Reference group: No plasma exchange therapy received.

^e^Reference group: No intra-epithelial infiltrates.
